# Aberrant Wnt Signaling in Leukemia

**DOI:** 10.3390/cancers8090078

**Published:** 2016-08-26

**Authors:** Frank J. T. Staal, Farbod Famili, Laura Garcia Perez, Karin Pike-Overzet

**Affiliations:** Department of Immunohematology and Blood Transfusion, Leiden University Medical Center, Albinusdreef 2, 2333 ZA Leiden, The Netherlands; f.famili@lumc.nl (F.F.); l.garcia@lumc.nl (L.G.P.); k.pike-overzet@lumc.nl (K.P.-O.)

**Keywords:** Wnt signaling, leukemic stem cell, leukemia, AML, CML, CLL, ALL, pathogenesis

## Abstract

The Wnt signaling pathway is essential in the development and homeostasis of blood and immune cells, but its exact role is still controversial and is the subject of intense research. The malignant counterpart of normal hematopoietic cells, leukemic (stem) cells, have hijacked the Wnt pathway for their self-renewal and proliferation. Here we review the multiple ways dysregulated Wnt signaling can contribute to leukemogenesis, both cell autonomously as well as by changes in the microenvironment.

## 1. Introduction: Hematopoiesis

**Figure 1:**
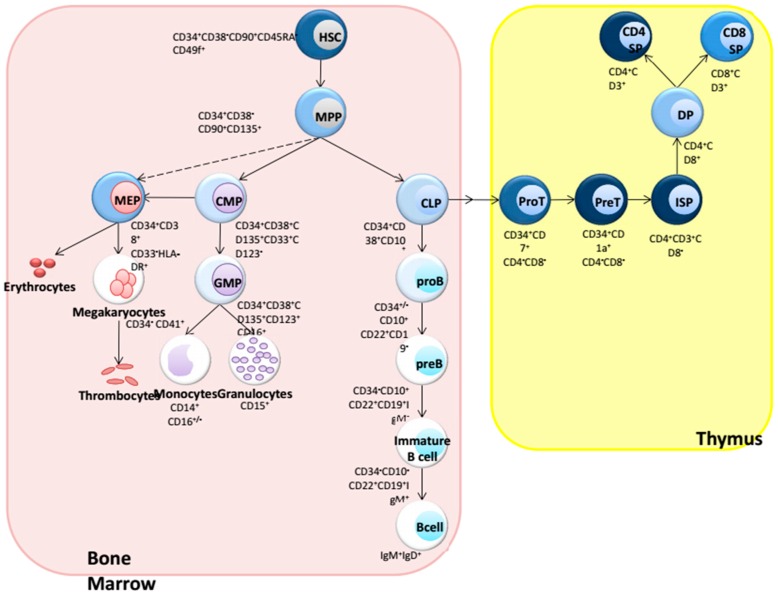
Normal human hematopoiesis. All blood cells are derived from a rare population of stem cells, which give rise to more lineage restricted progenitor cells that largely lack self-renewal. T-lymphocytes develop in the specialized microenvironment of the thymus. Wnt signaling is highest in the thymus, but also present in HSCs, CMP, CLP, and at low levels in pro B cells. The intensity of the blue color indicates the levels of Wnt signaling in the subpopulations depicted. All mature blood cells lack Wnt signaling, except a significant fraction of peripheral T cells. Data from Wnt signaling levels are derived from [15].

Blood cell formation, or hematopoiesis, is sustained by a rare population of multipotent cells that can be found in the adult bone marrow (BM) and that are responsible for the continuous production of blood cells throughout life [1]. These cells are commonly referred to as hematopoietic stem cells (HSCs) and are the origin of all blood cells, including platelets, erythrocytes, and all leukocytes. Research from many different laboratories has contributed to the phenotypic and functional characterization of HSCs, and nowadays the blood system constitutes the paradigm for understanding stem cell biology [2,3]. HSCs are located at the top of a hierarchy of lineage-specific progenitors that differentiate in an ordered fashion towards fully mature blood cells (Figure 1), thereby undergoing a stepwise loss of multi-lineage potential, and becoming progressively committed to a single hematopoietic lineage. HSCs have self-renewal type proliferation, which is lost upon differentiation towards committed lineages [4].

HSCs are functionally defined by their ability to mediate long-term (LT) multi-lineage repopulation after transplantation. The most stringent version of this operational definition requires that HSCs have to be serially transplantable in recipient animals while retaining both self-renewal and multi-lineage differentiation capacities [5]. As in stem cells from intestine, skin, and other solid organs, the Wnt signaling pathway plays an important role in the self-renewal of HSCs, although its role is more controversial [6,7]. The controversies on Wnt signaling as a self-renewal factor in hematopoiesis have recently been covered in depth [8].

As Wnt signaling plays an important role in normal hematopoiesis [6,9], it is often found in the malignant transformation of cells and its deregulation becomes more and more apparent in the malignancies of the hematopoietic system. The role of Wnt signaling in normal hematopoiesis and lymphocyte development has been covered extensively in a number of recent reviews [9,10,11]. We will therefore focus on the Wnt-pathway in the various leukemias and multiple myeloma and only briefly mention the role of Wnt-signaling in normal cell development, where it might contribute to the understanding of the derailed regulation of the Wnt pathway. We also will assume basic knowledge of the Wnt pathway and not extensively review the major properties of this pathway here. We instead refer to several excellent review articles that also schematically depict the Wnt pathways [12,13,14].

In short, the Wnt signaling cascade is often discerned into canonical or Wnt/β-catenin pathways and the non-canonical pathways. In the absence of Wnt ligands, cytoplasmic levels of β-catenin are kept very low through the action of a protein complex (the so-called destruction complex) that actively targets β-catenin for degradation. This complex is composed of two negative regulatory kinases, including Glycogen Synthase Kinase 3β (GSK-3β) and at least two anchor proteins that also function as tumor suppressor proteins, namely Axin1 or Axin2 and APC (adenomatous polyposis coli). APC and Axin function as negative regulators of the pathway by sequestering β-catenin in the cytoplasm. Activation of the pathway by Wnt leads to inactivation of the destruction complex, allowing build-up of the dephosphorylated form of β-catenin and its migration to the nucleus. In the nucleus, β-catenin binds to members of the TCF/LEF transcription factor family, thereby converting them from transcriptional repressors into transcriptional activators.

## 2. Hematological Malignancies

**Figure 2:**
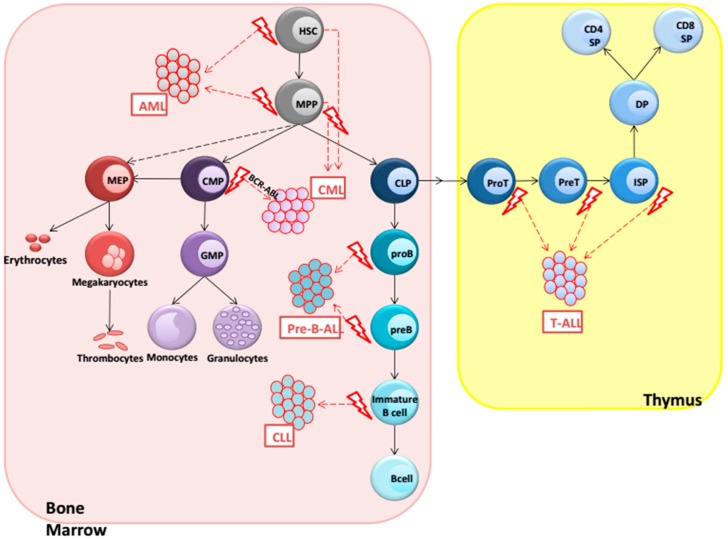
Leukemia development in humans. Leukemias are shown as malignant counterparts from normal hematopoietic cells. The figure is drawn to stress the similarities, not differences. For details on Wnt signaling in the various types of leukemia, see the main text.

Researchers in the field of hematologic malignancies currently aim to elucidate the exact role of aberrant Wnt signaling during pathogenesis. Before discussing the potential role of Wnt signals in leukemias, it is important to note that different hematologic malignancies arise at different anatomical locations, e.g., the bone marrow where all acute and chronic leukemias are thought to originate versus the thymus where T-cell acute lymphoblastic leukemia (T-ALL) originates, and that the various malignancies originate from different progenitor cells or from HSCs (see Figure 2). Both the microenvironment (bone marrow vs. thymus) and the cell of origin itself (stem cell, pro/pre-B cell, plasma cell, immature T cell) will greatly influence how normal Wnt signaling in these cells is deregulated and may eventually lead to malignant transformation. It is the interplay between extracellular signals, such as the production of Wnt proteins and Wnt antagonists in combination with the expression of intracellular components, such as the TCF/LEF factors and intracellular inhibitors, such as ICAT, in the progenitor cells themselves that will determine how the normally tightly controlled Wnt signaling levels [15] may tip the balance towards malignant transformation. Interactions with other signaling pathways and oncogenes further determine the type and malignant behaviors of the various leukemias. Therefore, careful analysis of the leukemic cells in combination with environmental factors may shed light on the exact role of Wnt signaling during leukemia development, as will be discussed per leukemia subtype in this review.

Finally, a note on nomenclature in mice vs. man. In animal models of leukemia, certain acute leukemias, i.e., those of B and T progenitors in the BM and thymus, are referred to as lymphomas, whereas in patients such malignancies are termed (acute) leukemias. In man, only malignancies in peripheral (secondary) lymphoid organs and other organs, i.e., skin, are termed lymphomas. For the sake of clarity we will use the latter terminology in this review.

## 3. AML (Acute Myeloid Leukemia)

Acute myeloid leukemia (AML) has many other names, including acute myelocytic leukemia, acute myelogenous leukemia, acute granulocytic leukemia, and acute non-lymphocytic leukemia. “Acute” means that this leukemia can progress quickly if not treated, and would probably be fatal within a few months. “Myeloid” refers to the cell type that is transformed, i.e., myeloid cells of the monocytic/granulocytic lineages. It is now well-established that AML is a clonal malignancy that originates in hematopoietic stem cells (HSCs) or very immature myeloid progenitor cells. It is the type of leukemia that is most closely related to normal HSCs. In AML, chromosomal translocations resulting in abnormal fusion proteins are frequently identified. Also, mutations that lead to FLT3 internal tandem duplications (ITDs), deletions of certain chromosomes, or abnormal karyotypes are all found, making the molecular causes of this disease highly heterogeneous.

The first studies describing a role for Wnt in AML showed that AML fusion proteins, such as AML1-ETO, PML-RARα, and PLZF-RARα specifically activated γ-catenin (also known as plakoglobin), the homologue of β-catenin, and lead to enhanced plakoglobin-LEF complexes, hence higher active Wnt signaling [16,17]. Also, in primary AML samples aberrant expression of β-catenin was found [18], and correlated with elevated levels of Wnt signaling as measured by Wnt-reporter constructs in primary AML samples. In AML blasts, the overexpression of γ-catenin appeared to correlate with β-catenin [19]. Interestingly, ectopic expression of γ-catenin in AML cells stabilizes β-catenin [19]. This stabilization of β-catenin might result from the fact that γ-catenin is less efficiently degraded in the Wnt destruction complex. Therefore, high levels of γ-catenin may obstruct the destruction complex in its task to degrade β-catenin by occupying the destruction complex, resulting in higher β-catenin levels.

Many studies demonstrate that epigenetic inactivation of Wnt pathway inhibitors by CpG island methylation provides an additional mechanism for the observed Wnt-pathway activity in AML leukemic cells. The methylation status of Wnt antagonists, such as SFRP-1, 3, 4, and DKK1, was shown to be responsible for the activation of the Wnt pathway in AML cells and correlated with poor prognosis [20,21,22,23,24,25]. Another natural antagonist of the canonical Wnt-pathway is the Wnt protein Wnt5a. This particular Wnt protein activates the non-canonical Wnt-pathway and mice that are hemizygous for Wnt5a develop myeloid leukemias [26]. Also in human samples, Wnt5a appeared to function as a tumor suppressor. In normal B cells, myeloid cells and CD34+ bone marrow cells Wnt5a transcripts were readily detectable. Analysis of several acute lymphoblastic leukemias (ALLs) showed that in samples of both B-ALL and AML the levels of Wnt5a were greatly reduced or completely absent [26]. One possible mechanism for the decreased levels of Wnt5a in AML cells was found to be analogous with a study on epigenetic regulation of Wnt5a in NK/T cell leukemias: promoter methylation of Wnt5a. Martin et al. state that the methylation status of Wnt5a is correlated with low expression of Wnt5a and a prognostic factor for poor prognosis in a subgroup of AML patients with intermediate-risk cytogenetics [22].

Important insights into the involvement of Wnt signaling in initiation and progression of AML blasts has come from mouse models for this disease. Both in a Mixed Lineage Leukemia (MLL) fusion protein induced AML mouse model (MLL-ENL) [27] and in a model using the retrovirally overexpressed oncogenes HOXA9 and MEIS1, the absence of β-catenin could prevent AML disease onset [28]. This indicates that although the first oncogenic event (MLL-fusion protein or overexpression of Hoxa9/Meis1) can induce the transformation of cells, thus generating pre-leukemic stem cells, dysregulated Wnt signaling is needed for the development and/or propagation towards full-blown leukemic stem cells (LSCs) in these mouse models of AML. This difference between pre-LSCs and LSCs was also confirmed in human primary leukemic AML cells [28]. Interestingly, also the anatomical location of pre-LSCs and LSCs was found to be different, in that pre-LSCs preferentially localize close to osteoblastic cells, whilst LSCs home further away from cells of the osteolineage cells [29]. Whether the difference in location is dictated by the Wnt signaling strength or whether the Wnt signaling levels are a result of the anatomical location remains elusive.

In clinical samples it was shown that activating mutations in FLT3 (FLT3 amplifications occur in about 30% of AML patients) were associated with high β-catenin levels [30] and that expression of total β-catenin, and more specifically non-phosphorylated nuclear β-catenin, correlated with a poor overall survival in AML patients [18,31]. The latter study shows that, although non-phosphorylated β-catenin could be found in the leukemic cells of all AML subtypes, it occurred most frequently in the M6 and M7 subtypes of the French-American-British (FAB) classification system. Hence, nuclear non-phosphorylated β-catenin was preferentially detected in erythroid and megakaryoblastic leukemia compared to other myeloid leukemias (M6–M7 vs. M0–M5). Recent insights based on genome wide expression profiling indicate that AML patients can be classified according to their WNT signature, which importantly has prognostic significance [32]. Additionally, new information indicates an important role for canonical Wnt signaling in the initiation of AML. Using mice that were deficient for the negative regulatory kinase GSK3β (and also its homolog GSK3α), Bhatia and coworkers showed that these mice have high Wnt signaling in their HSCs and develop a myeloplastic syndrome (MDS) similar to human MDS [33] which is seen as a precursor stage to full blown AML [34]. Deletion of β-catenin prevented the development of full AML and a similar gene signature as found in mice was also seen in human patients and carried prognostic significance [34].

Another interesting notion is that not only cell autonomous changes in the hematopoietic or leukemic stem cells are responsible for malignant transformation, but also that disruption of niche regulation by transformed hematopoietic cells, which may overexpress Wnt signaling or intrinsic stromal defects, is a collaborative factor in leukemogenesis. For instance, in AML, deregulation of the expression of certain Wnt ligands (i.e., WNT2B, WNT6, WNT10A, WNT10B) has been shown [35,36].

In conclusion, active Wnt signaling appears to play an important role in the propagation/acceleration of AML and has been shown to be an important secondary oncogenic event in mouse models of AML to transform pre-LSCs into LSCs. Based on these insights, new therapeutic opportunities have been explored by investigators who show that small-molecule WNT-pathway inhibitors, which inhibit the interaction between β-catenin and LEF1, selectively induce cell death in AML cell lines and primary AML blasts [37]. Recent studies in preclinical settings indicate the promise of WNT inhibition to treat AML [38,39,40], suggesting that targeted therapy of leukemic stem cells in AML may become possible.

An at first sight disparate result is the favorable outcome of cytogenetically normal AML patients that were associated with high LEF1 expression, reported by Metzeler and co-authors [41]. Other investigators have confirmed this important finding [42]. An explanation is that high LEF1 does not necessarily equate to high WNT signaling, as WNT-independent activities for LEF1 are described in other cellular processes, e.g., the function of LEF1 as a crucial transcription factor for granulocytic differentiation was shown to be independent of β-catenin [43]. It is also possible that Lef1, similar to Tcf1 in T-ALL [44], can function as a tumor suppressor gene, independent of β-catenin by acting as a repressor for proto-oncogenes. Careful analysis of LEF1 expression (and more specifically the short and long isoforms) in combination with the level of non-phosphorylated β-catenin on a per cell basis in different subtypes of AML patients may elucidate the WNT-dependent role of LEF1 in AML.

## 4. CML (Chronic Myeloid Leukemia)

Chronic myeloid leukemia is a myeloproliferative disorder characterized by the disease-causing translocation t(9;22), which gives rise to a shortened chromosome 22, the Philadelphia chromosome (Ph) [45,46] The translocation results in a fusion between the genes encoding the ABL tyrosine kinase and BCR, giving rise to the corresponding fusion protein: BCR-ABL. This fusion protein has constitutive tyrosine kinase activity and is considered causative in the disease [47,48,49,50], hence tyrosine kinase inhibitors such as imatinib is used for successful treatment of this disease.

Expression of the BCR-ABL fusion protein starts early in disease progression and was shown to correlate with high levels of active β-catenin during the blast crisis of CML [51]. BCR-ABL physically interacts with β-catenin, leading to its stability and enhanced nuclear localization [52]. BCR-ABL translocations are not only found in CML but also in 20%–30% of ALL cases and are associated with poor prognosis. In an elegant mouse model, the simultaneous development of both BCR-ABL induced CML and B-ALL could be investigated, and the researchers focused on the role of β-catenin in this process [52]. Normally, BCR-ABL-transduced cells cause a ratio of CML/ALL of 4:1 in the transplanted mice, however, when using Bcr-Abl-transduced cells lacking β-catenin (β-catenin −/−) this ratio was reversed (only 20% of the mice develop CML versus 80% of the mice that develop ALL) [52]. As CML and ALL arise from different cells of origin, and it has been shown that different cells of the hematopoietic system require different strengths of Wnt signaling during differentiation [15], these results suggest that myeolopoiesis is more dependent on canonical Wnt signaling as compared to B cell differentiation. This is in line with the fairly high levels of Wnt signaling detected in normal myeloid progenitors compared to B lymphocytes (almost zero) [15]. In addition, recent work using mice deficient for both Tcf1 and Lef1 showed that CML leukemic stem cells depend on these factors for initiation and propagation of the disease [53].

However, another possibility is the involvement of the non-canonical Wnt-pathway in the pathogenesis of CML and ALL as has recently been demonstrated by Gregory et al. [54]. In their quest to elucidate CML resistance towards tyrosine kinase inhibitors (TKI), they show the importance of the Wnt/Ca^2+^/NFAT pathway (a non-canonical Wnt pathway) in resistant CML cells and in particular the role of Fzd8. Resistant CML cells appear to be dependent on their Fzd8 expression for survival. This effect was suggested to be mediated via cytokine-induced Wnt5a production. Another explanation for the resistance of CML tumor cells towards TKIs is the selective survival of leukemic stem cells (LSCs) during treatment [55]. The survival of LSCs in a CML-mouse model was disrupted in β-catenin deficient mice, indicating a role for canonical Wnt signaling in TKI-resistance and occurrence of relapse in CML. Further evidence that β-catenin is crucial for LSC survival and maintenance in CML came from a recent study by Heidel et al. [56]. They showed that specific drug targeting of the β-catenin dependent Wnt-pathway in combination with TKI-treatment could completely eradicate the existence of LSCs in CML.

In conclusion, as the fusion protein BCR-ABL can actively modulate β-catenin levels in the cells, the most severely affected Wnt-pathway in CML is the canonical Wnt pathway. However, as recent studies show, in CML cells resistant to tyrosine kinase inhibitors the non-canonical Wnt-pathway might interfere when the BCR-ABL-mediated mechanism is inhibited. Therefore, novel therapeutics should not only be aimed at affecting the canonical Wnt-pathway but should also take into account the redundant effects of the non-canonical pathway, all in combination with tyrosine kinase inhibitors targeting BCR-ABL.

## 5. Precursor B-ALL

Acute lymphoblastic leukemia (ALL) is a rapidly progressive disease that involves immature lymphocytes, most often B-cell progenitors (80%) but also T-cell progenitors (20%) as malignant counterparts of thymocytes. The importance of the Wnt-pathway in normal B-cell development has been shown in two different settings; mice lacking Lef1 or Fzd9 show disrupted B-cell development with severely reduced B cell numbers [57,58]. The first role for Wnt signaling in pre-B ALL was shown in a subgroup that expresses E2A-PBX in a mouse model. These Pre-B-ALL cells were shown to produce Wnt16, and it is thought that this autocrine production of Wnt16 contributes to the development of pre-B-ALL [59]. Interestingly, only pre-B-ALL leukemias containing the t(1;19) chromosomal translocation resulting in the E2A-Pbx1 fusion protein were shown to express Wnt16. This suggests that Wnt16 is a target gene downstream of the fusion protein E2A-Pbx1 [59]. These results could not be confirmed by Nygren and co-workers [60,61]. They show that modulation of Wnt16 does not affect cell survival or proliferation. Instead they demonstrate that the high levels of β-catenin were located in the cell membrane alongside N-cadherin. Thus, they suggest that the high Wnt16 and β-catenin levels play a crucial role in the leukemia-stroma interaction instead of mediating high canonical Wnt signaling. Addition of Wnt3a to B-ALL cell lines and several B-ALL primary cells did show Wnt-reporter activity and was either shown to inhibit proliferation without affecting cell survival or to promote proliferation of ALL cells [61]. This indicates that an intact Wnt-pathway is present in B-ALL cells. Indeed, inhibition of B cell receptor signaling can interrupt Wnt signaling and thereby the survival of pre B-ALL cell lines [62].

## 6. CLL (Chronic Lymphocytic Leukemia)

Chronic lymphocytic leukemia is characterized by extended survival and accumulation of mature dysfunctional CD^5+^CD^19+^ B cells. Although it is a slowly developing disease, eventually malignant cells take over and cause reduced platelets and erythrocyte numbers, and lower numbers of T cells with associated clinical manifestations such as internal bleeding and infections. It is thought that the aberrant survival of these cells is mediated by mechanisms that evade apoptosis. Strikingly, WNT3 was highly and selectively expressed in CLL cells compared to other B cell malignancies (Diffuse Large B Cell Lymphoma (DLBCL) and follicular lymphoma) and normal T and B cells [63,64]. As Wnt3a has been shown to promote proliferation of mouse pro-B cells through a Lef1-dependent mechanism, it seems plausible that B cells in CLL exploit the same mechanism during CLL pathogenesis. Indeed, high levels of Lef1 (RNA and protein) were found in CLL cells but not in normal B cells [63,64]. Besides Lef1 and Wnt3, CLL cells also show high expression of the Wnt proteins: Wnt5b, Wnt6, Wnt10a, Wnt14, and Wnt16 as compared to normal B cells [64]. Further studies confirmed that aberrant activation of the canonical Wnt signaling is crucial for CLL pathogenesis. First of all, the addition of a specific inhibitor of the Lef-β-catenin interaction induced apoptosis in CLL cells whilst sparing the healthy cells [63]. Secondly, many studies have investigated the epigenetic regulation of the Wnt-pathway in CLL by studying the methylation status of Wnt-antagonists [65,66,67]. Alteration of the negative regulation of the Wnt-pathway by methylation of antagonists was shown for WIF1, DKK3, sFRP1, sFRP2, sFRP4, and sFRP5 in primary CLL cells.

Thus, active WNT signaling in CLL seems to be mediated via at least two mechanisms. First, the production and secretion of WNT proteins concomitant with the expression of WNT-receptors (such as FZD3 and LRP5/6), provides an autocrine loop. Second, the methylation status of the WNT-antagonists, explained by the high methylation status of antagonists as found in primary CLL samples, will contribute to higher WNT-activity in CLL cells.

An intriguing concept was recently explored by Gutierrez and co-workers. They reasoned that if the detrimentally high levels of LEF1 in CLL cells was closely correlated to the differentiation status of the cells, modulation of the CLL cells could affect disease progression [68]. Indeed, by inducing the further differentiation of CLL cells into immunoglobulin secreting cells, and by using the TLR9 agonist CpG, they demonstrated impaired cellular survival and lower WNT-activity in the leukemic cells. Finally, WNT5A levels associate, independent of IGHV status, with the clinically worst CLL subgroups characterized by dysfunctional p53 and mutated SF3B. WNT5a may allow CLL cells to escape from inhibitory signals normally found in the BM environment [69].

## 7. T-ALL (T-Cell Acute Lymphoblastic Leukemia)

Leukemic cells in T-cell Acute Lymphoblastic Leukemia are characterized by an immature phenotype of developing T lymphocytes in the thymus. Hence, pathways involved in the development of T cells [70,71] are often deregulated in T-ALL, as has been shown for Wnt and Notch signaling [72,73,74,75]. The crucial importance of Notch1 in T-ALL development is underlined, as most of the mouse models showing development of leukemia due to the absence of either E2a, Ikaros, or Tcf are accompanied by activating mutations in Notch1 [76]. Although these mutations were not the leukemia-initiating event, the likelihood of additional mutations to occur in Notch1 appears to be very high and accelerates lymphoma development. It has been shown that over 50% of human T-ALL samples contain activating mutations in the NOTCH1 gene [74]. Whilst Tcf-1 was recently reported to be a downstream target of NOTCH1 [77], it seems likely that mutations in TCF1 can also occur in T-ALL patients as secondary hits after an initial mutation in NOTCH1. In T-ALL mouse studies, investigators have specifically overexpressed crucial proteins in either the Wnt or the Notch pathway (non-degradable form of β-catenin or constitutive expression of intracellular Notch1 (ICN)) [78,79,80]. These studies demonstrate that continuous activation of either the Wnt or the Notch pathway result in the development of highly aggressive leukemias [78,80]. Of interest, the study by Guo et al., that uses the constitutively active form of β-catenin, reports leukemia development without occurrence of Notch1 mutations [78]. Since in all other studies Notch1 mutations are frequently reported, this is a remarkable observation, although studies with human patients also show that high WNT levels occur in T-ALL cases without activating mutations in NOTCH1 [81]. This suggests that aberrant WNT signaling might not just be an additional mutation in pre leukemic T-ALL cells, but similar to NOTCH signaling can be the leukemia initiating event.

Of special interest are two recent studies that show a clear tumor suppressor role for Tcf1 in T-ALL development [44,82]. Both studies show that mice deficient for Tcf1 are highly susceptible to develop leukemias. The observed leukemias had a heterogeneous pattern of leukemia formation, which is expected as Tcf-deficiency leads to several incomplete and consecutive T-cell blocks in development [44]. Remarkable was the high expression of Lef1 in these leukemias (and Id2 in the DN3 lymphomas in the study by Yu et al. [82]). Both studies show that Tcf1 normally acts a suppressor of Lef1 protein levels in the thymus. Upon deletion of Tcf1, Lef1 protein levels become deregulated in all developmentally blocked thymic subsets, resulting in abnormally high levels of the long isoform of Lef1, predisposing the thymocytes towards leukemic transformation. The question remains whether this Lef-mediated oncogenic effect, due to the absence of Tcf1, is a Wnt-driven process or not [83]. The study by Tiemessen et al. demonstrated elevated Wnt signaling activity in the Tcf1-deficient tumors by crossing the Tcf1-deficient mice to a Wnt-reporter mouse [44]. As the study by Yu et al. could not demonstrate this [82], there might be a possibility that high Lef1 levels mediate leukemia formation in an additional Wnt-independent way. Crossing of the Tcf1-deficient mouse to an inducible Lef1-knockout mouse confirmed the redundancy of both factors as there was an almost complete lack of T-cell development. However, in the double knock-out (Tcf/Lef) mice some leukemia formation (2 out of 13 mice) was still reported [82]. This may suggest the existence of an additional Wnt-pathway-independent mechanism causing these lymphomas. However, a key role for Lef1 was also found in a mouse model that deregulates the Notch pathway by ectopic expression of the intracellular domain of Notch1 [84]. This study showed that T-cell lymphoma lines require high levels of Lef1 for their survival. Hence, deregulation of Lef1 expression, either via lack of the tumor suppressor Tcf1 or via increasing of the Notch pathway accelerates lymphomagenesis. Also, in human cases of precursor T-ALL, aberrantly active WNT signaling has been reported [81]. In addition, the gene expression profile data of Tcf1-deficient lymphomas in the study by Tiemessen et al. show high levels of Mef2c, which has been recently identified as a subtype of T-ALL in pediatric patients [85]. These data indicate that Tcf1 also acts as a tumor suppressor in certain subtypes of pediatric T-ALL (ETP T-ALL and MEF2C-positive T-ALL).

In contrast to AML, leukemic stem cells in ALL have long been controversial, although based on deregulated Wnt signaling such stem cells have long been predicted [73]. Recent important studies indicate that T-ALL stem cells exist and are highly dependent on Wnt signaling, similar to AML stem cells [86]. Active Wnt signaling was shown to be restricted to minor subpopulations within bulk tumors, and these Wnt-active subsets were highly enriched for T-ALL stem cells. Importantly, the reduction in β-catenin levels severely reduces leukemic stem cell frequency. In addition, in these stem cells another stem cell pathway, namely the hypoxia pathway, was activated. Deletion of the hypoxia response mediator, Hif1α, severely reduced stem cell frequency. Of note, the deletion of β-catenin or Hif1α did not impair the growth or viability of bulk tumor cells, suggesting that elements of the Wnt and Hif pathways specifically support leukemic stem cells [86].

## 8. Conclusions

For all hematologic malignancies discussed in this review, Wnt signaling (canonical and non-canonical) appears to be involved at a certain phase during pathogenesis. There seem to be at least two different stages at which dysregulation of Wnt signaling plays a role in leukemogenesis. First, at the initiation phase of the disease, elevated Wnt signaling could be the crucial factor to progress from a pre-LSC into an LSC (as for AML) and as has been recently discovered for T-ALL where the absence of the Wnt-nuclear factor Tcf1 appears to predispose to T-ALL development. However, also in progression of established disease, as described for CML, ectopically activated Wnt signaling is important. Hence, depending on the differentiation status of the cell and or the location of the leukemia-initiating cells in its microenvironment (niche), Wnt signaling plays different roles in leukemogenesis.

There seem to be at least five different mechanisms involved in the observed deregulation of Wnt signaling in leukemias. First, aberrant levels of Wnt proteins (and/or Wnt antagonists) can be secreted by tumor cells themselves and/or the microenvironment, frequently reported as an autocrine feedback loop of the tumor cells. Second, the sensitivity of the tumor cells towards the Wnt proteins (and antagonists) might be altered (for example changes in Fzd, Ror, or LRP expression). As a third mechanism, epigenetic changes are repeatedly reported in most types of leukemias. Both methylation of Wnt antagonists (thereby interfering with their suppressive function) and of the Wnt5a promoter (resulting in low levels of Wnt5a, which acts a tumor suppressor) have been found. Fourth, activating mutations in β-catenin or inactivating mutations in APC or Axin have been described in ALL, akin to well-known activating mutations in colon carcinomas. And fifth, the balance of the Tcf/Lef factors within a tumor cell appears to be an important determining factor whether Wnt signaling derails during development (see also a summary in Table 1).

Needless to say, these alterations in Wnt signaling offer possibilities for therapeutic intervention, especially because the levels of Wnt signaling in hematological malignancies are significantly higher than their normal counterparts [29,87]. In addition, when combined with treatments for classical abnormalities (targeted by tyrosine kinase inhibitors in CML (imatinib, dasatinib) [47,88], Notch inhibitors in T-ALL [89], and BTK inhibitors in precursor B-ALL (ibrutinib) [90]), the necessary dosage of Wnt inhibitors is expected to be lower as one can envision combination therapies targeting Wnt signaling together with the other genetic alterations to work synergistically. In this way the fundamental knowledge about regulation of normal and malignant development can be rapidly translated into clinical practice.

## Figures and Tables

**Table 1:** Summary of mechanism underlying deregulated Wnt signaling in leukemia.

Possible Mechanism	Hematological Disorder	References
Wnt protein secretion by tumor cells or microenvironment	AML: tumor cells produce Wnt2B, Wnt6, Wnt 10A, Wnt10B	[35,36]
	CML: human BM MSC cells secrete Wn1, Wnt2B, Wnt3, Wtn5a, Wtn5B, Wnt6, Wnt8b, Wnt16	[91,92]
	B-ALL: tumor cells produce Wnt16b	[61,93]
	CLL: tumor cells express Wnt3a and Wnt5B, Wnt6, Wnt10A, Wnt14 and Wtn16	[69,94]
Responsiveness of tumor cells to Wnt-signaling	CML: TKI-resistant cells have a high Fzd8 expression	[52]
	CLL: tumor cells express Fzd3 and LRP5/LRP6 and Ror1	[63,64,95]
Epigenetic changes (aberrant methylation of Wnt antagonists or Wnt5a)	AML: methylation of sFRP-1, 3, 4, and DKK1, or Wnt5a	[20,22,23,24,32]
	B-ALL: methylation of DKK3	[96]
	T-ALL: inappropriate methylation of Wnt5a	[97]
	CLL: methylation of Wif1, DKK3, sFRP-1, 2, 4, and 5	[65]
Activating mutations in β-catenin or inactivating mutations in APC or Axin	AML and ALL: inactivating mutations in Axin1 and APC	[16,17,19,98,99]
	T-ALL: activating mutations in β-catenin, loss of TCF7 tumor suppressor activity	[81,82]
Balance of Tcf/Lef factors in tumor cells	AML: high Lef levels	[41,42]
	CML: Tcf/Lef factors positiviely regulate ABCB1	[100]
	B-ALL: disbalance of Tcf and Lef levels in tumor cells	[60]
	T-ALL: high Lef levels; Tcf1 is tumor suppressor gene	[44,101]
	CLL: high Lef levels	[64]
